# Endoscopic Right Lobectomy Axillary-Breast Approach: A Report of Two Cases

**DOI:** 10.1155/2010/958764

**Published:** 2010-12-28

**Authors:** Nina Irawati

**Affiliations:** Awal Bros Hospital, Batam, Riau Island, Indonesia

## Abstract

*Background*. We reported our two initial experiences in the treatment of thyroid disease with endoscopic thyroidectomy. Minimally invasive video-assisted technique (MIVAT) was initially introduced by Miccoli. The modification was made by using axillary and breast technique. *Method*. Two young women patients, with 4 and 5 cm right lobe thyroid disease suspected to be benign. From physical examination, sonography, and FNAB findings, the two cases were categorized as benign. We performed endoscopic right lobectomy through incision of 5–10 mm on axillary line and breast using CO2 insufflation. *Result*. Duration of first operation was 300 minutes and the second one was 120 minutes, with minimal blood loss and no major complication. Patients were discharged 24 hours after operation. Cosmetic result was excellent. Postoperative complications were shoulder discomfort and neck swelling. *Conclusion*. We reported two cases of endoscopic right lobectomy as a safe, reproducible technique with an indication in a minority of patients candidates for thyroidectomy and is characterized by less postoperative discomfort.

## 1. Introduction

Neck surgery is one of the newest and most interesting application of minimally invasive surgery technique in thyroid surgery, particularly with regard to eliminating the unattractive scars [1, 2]. 

It is well known that conventional thyroidectomy allows for prompt postoperative recovery. In some clinical settings, it is performed as an outpatient procedure. Findings have shown that video-assisted and endoscopic procedures for thyroid surgery have some advantages over conventional surgery in term of cosmetic result and postoperative recovery. These results support the development of endoscopic and video-assisted thyroid surgery. It should be emphasized that these procedures are technically demanding and require a surgical team skilled in both endocrine and endoscopic surgery. This is particularly true for some endoscopic techniques such as endoscopic thyroidectomy by breast or axillary approach. The endoscopic and video-assisted procedure requires a significant learning period, which can be time consuming especially at the beginning of a surgeon's experience [3].

Minimal-access thyroid surgery was conceived primarily in Europe and Asia [1]. Endoscopic neck surgery for the parathyroid and thyroid was developed by Gagner and Huscher in 1996 and 1997, respectively. Since then, various methods including axillary, breast, and anterior chest approaches have been introduced by many surgeons. The use of endoscopic for complete thyroidectomy has been viewed with concern, although many surgeons have regarded benign thyroid disease as an indication for endoscopic surgery [4–7].

**Case Illustration I**

This case is a 32-year-old women with lump on right anterior neck for the last year. From history taken and clinical examination, the lump had benign sign; its size was 5 cm without regional lymphadenopathy. Laboratory findings were within normal limit. Sonography preoperatively showed a 5-cm hypoechoic solid mass with smooth and thick peripheral halo, suggestive of adenoma of thyroid without any lymphadenopathy. FNAC revealed follicular lesion. We performed right lobectomy endoscopically with the lack of frozen section facility. Final pathologic result was follicular neoplasm. Further treatment is needed, and she is now prepared for completion thyroidectomy endoscopically.

**Case Illustration II**

This case is a 34-year-old women with neck swelling for the past 6 months. Clinically, the lump was from thyroid region with benign symptoms. The size was 4 cm, with no cervical lymphadenopathy observed. Laboratory finding within normal limit. Sonography and FNAB findings suggested a benign cyst. We performed right lobectomy endoscopically. Pathologic result was benign thyroid cyst. No further treatment was needed.

## 2. Method

In our initial two case reports, we used the axillary-breast approach to perform endoscopic right lobectomy. Under general anesthesia, those patients were placed in the supine position with the neck moderately extended. The port sites were identified. At first, 10-mm longitudinal incision was made at anterior axillary region. Then 5 mm incision circumareolar and shoulder. Then a vascular clamp was used to create the preliminary subfascial space. A 10-mm port was placed as the optical port. The operating space was maintained with CO2 insufflation at a gas pressure of 10-11 mm Hg. A 10 mm 0^0^ endoscope was inserted. Under its guidance, we inserted other 5 mm port, respectively. The subcutaneous tunnel was further enlarged with bipolar and hook equipment. The lateral border of sternocleidomastoideus was dissected and omohyoid was moved upward. The thyroid gland was exposed. The inferior and superior thyroid arteries were divided using harmonic scalpel. The parathyroid gland and recurrent laryngeal nerve (RLN) were routinely identified and preserved. The gland was dissected by harmonic scalpel as well.

## 3. Result

The duration of first operation was 300 minutes and compared to the first, we performed our second operation slightly faster (120 minutes). Intraoperatively, the amount of bleeding was 75 cc and lesser in our second operation (30 cc). Such complications that we observed postoperatively including shoulder discomfort and neck swelling disappeared after 7–10 days. Postoperative seroma, hypocalcemia, and recurrent laryngeal nerve paralysis were not seen (proven by normal calcium level examination and mobile cords by laryngoscope postoperatively). All patients were discharged in a good condition.

## 4. Discussion

The history began with the initial experience conducted with MIVAP (minimally invasive video-assisted parathyroidectomy) leading some authors to perform the same surgical approach for thyroidectomy. The first idea that motivated MIVAT (minimally invasive video-assisted thyroidectomy) was the better cosmetic result (an incision of 1,5–2 cm). Miccoli introduced this technique as a three-part procedure starting with an open technique, then followed by endoscopic component and then completed in an open fashion. According to some data in the literature, any surgeon approaching the MIVAT technique must carefully consider that at the beginning of his or her experience, the procedure will be significantly longer than the standard operation. Since then, endoscopic thyroidectomy has been divided into two types, with CO2 insufflation or gasless. Others classified it as videoassisted and total endoscopic [1, 7, 8].

Total endoscopic thyroidectomy is a more sophisticated variation of minimally invasive thyroid surgery. Using special instrument and technique, part or all of the thyroid gland can be removed through small puncture site, avoiding any incision on the neck whatsoever. In this technique, the skin overlying the collarbone is lifted from the underlying muscle and laparoscopic techniques are used to create a working space [8].

Various approaches have been devised and improved further to fulfill this goal, mainly including the cervical approach, anterior chest approach, and axillary and breast approaches. However, none of these approaches is exclusively advantageous and universally accepted. The cervical approach and anterior chest approach are minimally invasive, but not cosmetically excellent. The axillary and breast approaches have maximized cosmesis, but meanwhile cause much invasiveness. Furthermore, the axillary approach is not suitable for bilateral manipulation and even more technically challenging with abnormal anatomic vision. Therefore, an axillary-bilateral-breast approach (ABBA) has been developed, which is actually a combination of the procedures. In comparison, ABBA permits bilateral exploration, more space for instrument use, and the removal of larger nodule. With this technique, the mean surgical time was 188 minutes, mean blood loss was 53 mL, and mean hospital stay was 3,3 days. BABA (bilateral-axillary-breast approach) was introduced later and was claimed to be easily applied for thyroid cancer as well. The difference between these techniques is based on the type of the port. ABBA technique involves the use of two circumareolar ports and a single axillary port, whereas BABA is performed by using two circumareolar ports and two axillary ports [3, 5–7, 9]. This technique now is even improved by using Da vinci robotic system by Lee et al. [6] which is useful in identification of anatomy and dissection during surgery. The endowrist function of the instrument is beneficial in doing complex tasks in difficult areas with limited access. The mean operating time without robotic system was 165 minute and with robotic system was 218,3 minute, but there is tendency towards a decrease in operation time. They also perform central node dissection and limited lateral node picking. Another technique is using axillary-breast approach which can be applied even for bilateral procedure, including central neck dissection. 

The first endoscopic surgery, which was performed transcervically, was employed to treat a 3-mm moderately differentiated papillary microcarcinoma with focal capsular invasion in 1997. The use of a transcervical approach results in small operative scars in the neck [4]. After this attempt, Ohgami et al. [10] performed endoscopy via breast approach for thyroid adenoma 5–7 cm in diameter. Yamamoto et al. [11] applied endoscopy by breast approach for Graves' disease patients. Ikeda et al. [12] applied anterior chest and axillary approach for follicular tumors, Graves' disease, and papillary microcarcinoma.

Generally, endoscopic thyroid surgery has been thought to be appropriate for benign thyroid disease. First, it was indicated for nodule not more than 3 cm, benign or low-grade follicular lesion, and papillary carcinoma, with contraindications for previous neck surgery, large goiter, local metastases, previous neck irradiation, thyroiditis, and hyperthyroidism [13]. These indications during the development of the technique then slightly changed. Some even can perform them for nodule more than 5 cm, for Graves' disease, and thyroiditis [4]. The role of endoscopy for carcinoma is still in debate. In other areas of oncologic surgery, such as gastric or colorectal carcinoma, minimally invasive laparoscopic surgery has been established through clinical experience and technical development. Similarly, endoscopic thyroid surgery can be used for malignant thyroid disease. BABA and axillary-breast technique can be applied as an appropriate method for treating thyroid malignancies [4, 6]. Kitano et al. [14] reported treatment of thyroid cancer with anterior chest approach endoscopic surgery. The indications are as follows: age less than 45 years, tumor size smaller than 2 cm, and no evidence of lymph node metastases or local invasion. Miccoli et al. [15] showed that the completeness obtained with MIVAT for thyroid carcinoma not exceeding 3,5 cm in diameter is similar to that obtained with open surgery. As experience accumulates and more techniques are developed, the indication in cases of thyroid malignancy can be expanded [4, 6].

Postoperative complications include hypocalcemia, recurrent laryngeus nerve (RLN) paralysis, bleeding, infection, and pain [4, 5]. Others as a complication of using CO2 insufflation are hypercapnia, subcutaneous emphysema, and severe tachycardia [5, 16]. Gottlieb et al. [17] reported severe increases in PaCO2, subcutaneous emphysema, and severe tachycardia when applying insufflations at relatively high pressure (15–20 mm Hg), whereas Ochiai et al. [16] and Ohgami et al. [10] reported only minimal emphysema with the use of low pressure CO2 insufflation (6 mm Hg). Our technique used 10-11 mm Hg insufflations of CO2 without severe complication. The study of the appropriate pressure that should be used is still under observation.

 According to the literature, the conversion rate varies from 0–13%. The reasons include malignant histological result, bleeding, difficulty of dissection, size of nodule, and thyroiditis. In some reports, 5–11% of patients even required a second operation for definitive malignant pathological result [6].

We performed our cases using axillary-breast approach. From our opinion, this technique is safe and feasible to perform. It provides excellent view of vital structure and has advantage over open method cosmetically. The minimal amount of bleeding and mild postoperative complications have added another advantages. Even though multiple studies have demonstrated the variety of endoscopic thyroidectomy, there are some fields which need further observation such as appropriate pressure and whether this technique is oncologically safe for malignant case.

## 5. Conclusions

Since its introduction and establishment in 1997, endoscopic thyroidectomy has become a standard procedure. Thus this procedure will provide another surgical choice for patients with thyroid tumors and carcinoma.

## Figures and Tables

**Figure 1:**
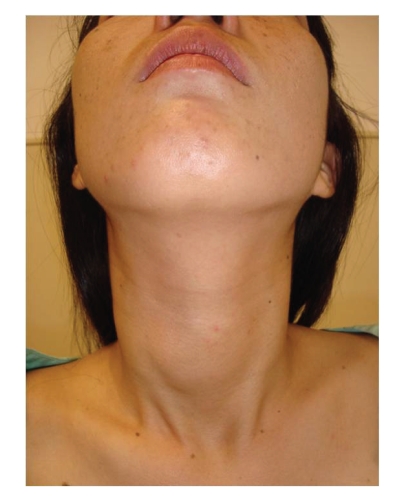
Local condition preoperatively.

**Figure 2:**
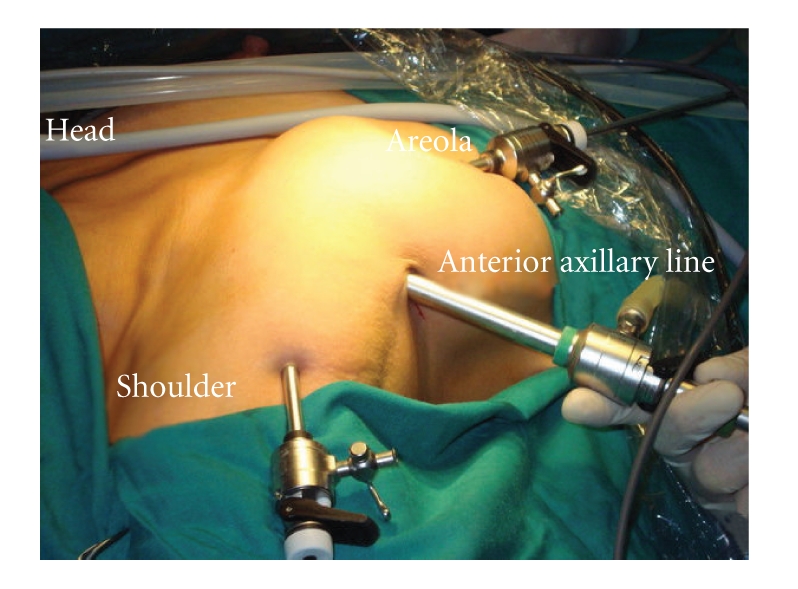
Axillary-breast technique.

**Figure 3:**
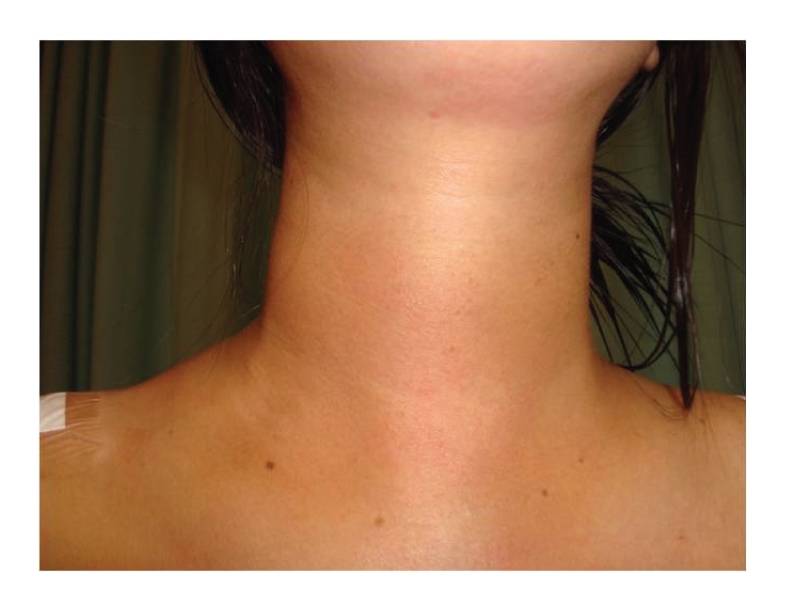
Local condition postoperative.

## References

[1] El-Labban GM (2010). Minimally invasive video-assisted thyroidectomy : a single-blinded, randomized trial. *Internet Journal of Surgery*.

[2] Bellantone R, Lombardi CP, Bossola M (2002). Video-assisted vs conventional thyroid lobectomy: a randomized trial. *Archives of Surgery*.

[3] Gal I, Solymosi T, Szabo Z, Balint A, Bolgar G (2008). Minimally invasive video-assisted thyroidectomy and conventional thyroidectomy: a prospective randomized study. *Surgical Endoscopy*.

[4] Chung YS, Choe JH, Kang KH (2007). Endoscopic thyroidectomy for thyroid malignancies: comparison with conventional open thyroidectomy. *World Journal of Surgery*.

[5] Park YL, Han WK, Bae WG (2003). 100 Cases of endoscopic thyroidectomy: breast approach. *Surgical Laparoscopy, Endoscopy and Percutaneous Techniques*.

[6] Lee KE, Rao J, Youn YK (2009). Endoscopic thyroidectomy with the da vinci robot system using the bilateral axillary breast approach (BABA) technique: our initial experience. *Surgical Laparoscopy, Endoscopy and Percutaneous Techniques*.

[7] Wang YL, Zhang GY, Wang L, Wang KEX, Hu SY (2009). Endoscopic thyroidectomy by a modified anterior chest approach: a single institution’s 5-year experience. *Minimally Invasive Therapy and Allied Technologies*.

[8] Minimally Invasive Thyroid Surgery http://www.thyroidectomy.com.

[9] Bärlehner E, Benhidjeb T (2008). Cervical scarless endoscopic thyroidectomy: axillo-bilateral-breast approach (ABBA). *Surgical Endoscopy*.

[10] Ohgami M, Ishii S, Arisawa Y (2002). Scarless endoscopic thyroidectomy: breast approach for better cosmesis. *Surgical Laparoscopy, Endoscopy and Percutaneous Techniques*.

[11] Yamamoto M, Sasaki A, Asahi H (2001). Endoscopic subtotal thyroidectomy for patients with Graves’ disease. *Surgery Today*.

[12] Ikeda Y, Takami H, Sasaki Y, Takayama J, Niimi M, Kan S (2002). Comparative study of thyroidectomies: endoscopic surgery vs conventional open surgery. *Surgical Endoscopy*.

[13] Del Rio P, Sommaruga L, Ferreri G, Arcuri MF, Sianesi M (2006). Preliminary experience in minimally invasive videoassisted thyroidectomy (MIVAT). *Acta Biomedica de l’Ateneo Parmense*.

[14] Kitano H, Fujimura M, Kinoshita T, Kataoka H, Hirano M, Kitajima K (2002). Endoscopic thyroid resection using cutaneous elevation in lieu of insufflation: technical considerations and review of an open series. *Surgical Endoscopy*.

[15] Miccoli P, Berti P, Raffaelli M, Materazzi G, Baldacci S, Rossi G (2001). Comparison between minimally invasive video-assisted thyroidectomy and conventional thyroidectomy: a prospective randomized study. *Surgery*.

[16] Ochiai R, Takeda J, Noguchi J, Ohgami M, Ishii S (2000). Subcutaneous carbon dioxide insufflation does not cause hypercarbia during endoscopic thyroidectomy. *Anesthesia and Analgesia*.

[17] Gottlieb A, Sprung J, Zheng XM, Gagner M (1997). Massive subcutaneous emphysema and severe hypercarbia in a patient during endoscopic transcervical parathyroidectomy using carbon dioxide insufflation. *Anesthesia and Analgesia*.

